# Developing a person-centred care environment aiming to enhance the autonomy of nursing home residents with physical impairments, a descriptive study

**DOI:** 10.1186/s12877-023-04434-8

**Published:** 2023-11-15

**Authors:** Jolande van Loon, Meriam Janssen, Bienke Janssen, Ietje de Rooij, Katrien Luijkx

**Affiliations:** 1 Department of Tranzo, School of Social and Behavioural Sciences, Tilburg University, Tranzo, Post Box 90153, Tilburg, 5000 LE The Netherlands; 2 De Wever, Organisation for Elderly Care, Post Box 1173, Tilburg, 5004 BD The Netherlands; 3 School of People and Health, Fontys University of Applied Sciences, Post Box 347, Eindhoven, 5600 AH The Netherlands

**Keywords:** Autonomy, Care environment, Document study, Nursing homes, Older adults with physical impairments, Organisational policies, Person-centred practice, Semi-structured interviews

## Abstract

**Background:**

Enhancing autonomy is important within the context of the care environment in nursing homes. A nursing home is a place for older adults with physical impairments, who need assistance, to live and where staff work who help them to exercise autonomy. Previous research shows that older adults and staff are influenced by the care environment to apply autonomy-enhancing activities. Therefore, organisational policies regarding the care environment seem promising for enhancing autonomy. The aim is to gain a deeper insight into the development and implementation of organisational policies aimed to enhance the autonomy of older adults with physical impairments.

**Methods:**

A qualitative descriptive design was chosen, using two methods. A document study was conducted on the policies, plans and proceedings in two care organisations. Moreover, interviews were conducted with 17 stakeholders involved in the policies, such as managers and members of the client council. The fragments of the 137 documents and 17 verbatim transcripts were coded and deductively categorised into the seven aspects (i.e., power-sharing, supportive organisational systems, appropriate skill mix, potential for innovation and risk-taking, the physical environment, effective staff relationships and shared decision-making systems) of the key domain care environment, as defined in the person-centred practice (PCP) framework developed by McCormack and McCance.

**Results:**

The aspect of power-sharing was used the most in the policies of the two participating organisations. The organisations expected much from the implementation of indirect interventions, such as access to the electronic care plan for residents and the development of staff towards self-managing teams. Less attention was paid to interventions in the physical environment, such as the interior of the building and privacy, and the collaboration processes between staff.

**Conclusions:**

The PCP framework poses that all aspects of the key domain care environment are important to develop a person-centred practice. This is not yet the case in practice and the authors therefore recommend using all seven aspects of the care environment in a balanced combination with the other key domains of the PCP framework to achieve person-centred practice and as a result the enhancement of the autonomy of nursing home residents with physical impairments.

## Introduction

### Background

Older adults with physical impairments due to chronical health conditions or old age (hereafter referred to as older adults with physical impairments), who need 24-h care and intensive help with activities of daily living (ADL) often move to a nursing home. This move to a nursing home contributes to feelings of dependency and challenges the older adult to find a way to be able to live their life as before and as preferred. Being able to maintain autonomy is important for older adults who live in a nursing home. Generally speaking, older adults with physical impairments are able to make decisions on how they want to live their lives. However, they are often hindered in terms of executing these decisions due to the underlying physical conditions that made them move to the nursing home. Tensions between freedom and best intentions of staff, autonomy and dependence, individual preferences and the pressures of collective care, can be present [1].

According to the literature, autonomy can be described as the capacity to affect the environment, irrespective of having executional autonomy, to live the kind of life someone desires to live in the face of diminishing social, physical and/or cognitive resources and dependency, and autonomy develops in relationships [2]. However, autonomy should also be considered from a broader perspective. Both older adults [3] and staff [4] indicate that they are influenced by the care environment of the nursing home to apply effective mechanisms and activities to enhance autonomy. Schedules, checklists, and protocols can for example, be helpful to organise care, but if they prevail above the persons, i.e., the resident and staff, they can hinder autonomy.

Person-centred care is seen as a way to enhance autonomy i.e. when caregivers consciously engage in the care for older adults who are striving to live the life they desire to live, autonomy can be maintained [5]. The board managers of nursing homes recognise the importance of autonomy and aim to enhance the autonomy of older adults and therefore they seek to develop and implement autonomy enhancing policies [6].

There is little research done about how the care environment is shaped by organisational policies with the aim to enhance autonomy for older adults with physical impairments. In one study, two mechanisms i.e., choice enhancing and control enhancing policies were found to strengthen the autonomy of residents [7]. Results of that study show that organisations mostly used choice enhancing policies aimed to give residents choice in daily routines such as the time to go to bed and what and when to eat. This policy seemed to be related to higher feelings of autonomy in residents. One intervention to strengthen autonomy, related to enhancing control at the organisational level, was found [8]. However, this study did focus on autonomy related to resident participation in formal decision making, rather than on improving autonomy in day-to-day care. The current study will concentrate on enhancing autonomy in the care environment from a wider perspective.

### Aim

The objective of this study is to gain a deeper insight into the development and implementation of organisational policies aimed to enhance the autonomy of older adults with physical impairments in nursing homes. This will be done by answering three research questions (RQs), i.e., RQ1; which policy is developed by board managers of nursing homes with the aim to enhance autonomy, RQ2; what is reported in the proceedings and evaluation of this policy and RQ3; what are the perspectives and experiences of stakeholders involved in the implementation of the policy in daily practice?

## Theoretic framework

**Fig. 1:**
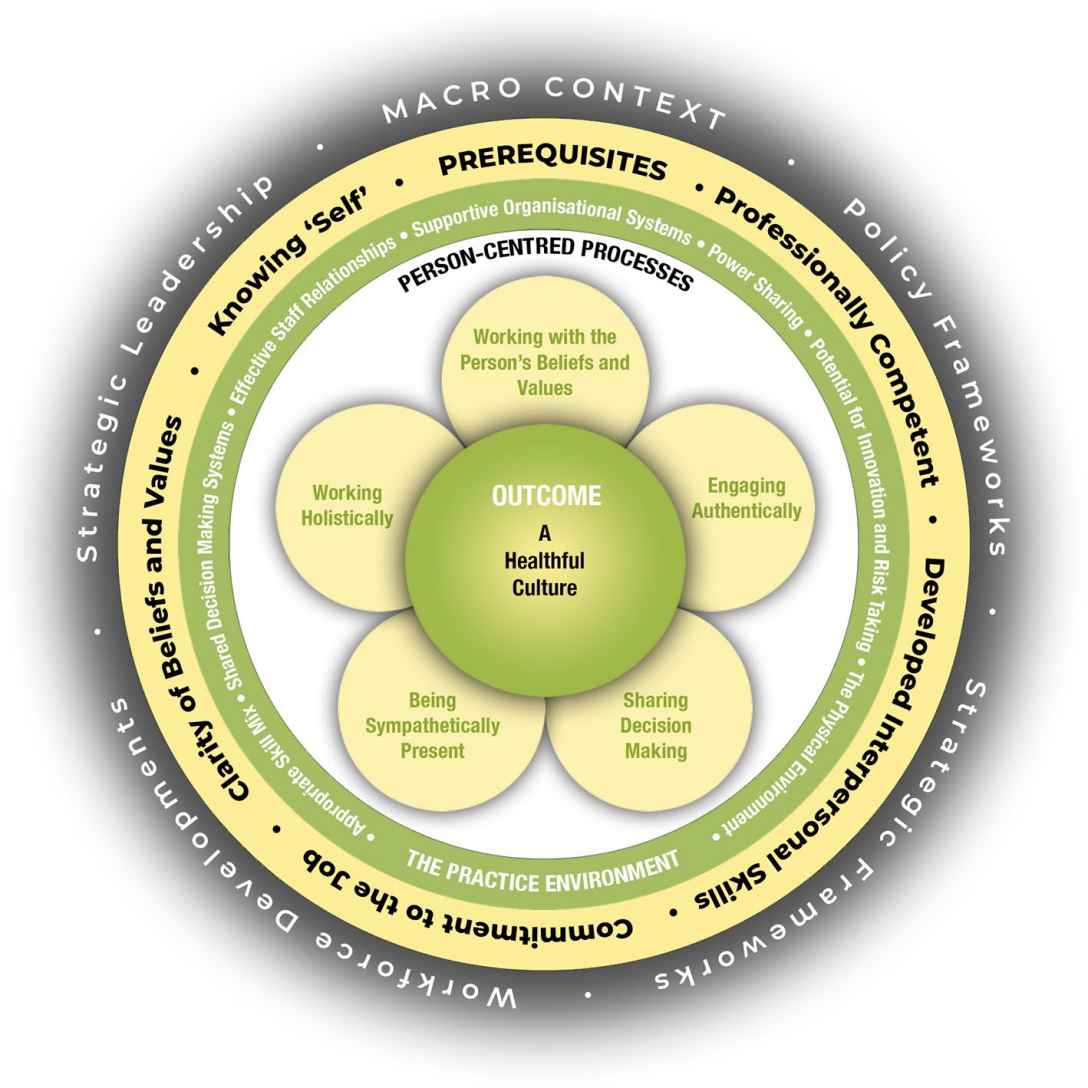
Person-centred Practice framework. Retrieved from The Centre for Person-centred Research practice (CPCPR) of Queen Margaret University Edinburgh. Reused with permission from McCormack & McCance

As previously stated, person-centred care is considered to enhance and respect autonomy of older adults living in a nursing home [5]. Because different interpretations of person-centred care are used in the literature, the authors choose for an evidence-based framework. McCormack and McCance [9] present a Person-centred Practice (PCP) framework which offers evidence based aspects that are important to enhance autonomy. Three key domains are described in this framework: i.e., person-centred processes, the care environment, and the prerequisites of staff. The PCP framework is presented in Fig. 1.

The care environment, in Fig. 1 named practice environment, is situated between the person-centred processes and the prerequisites of staff. It can either function as a facilitator or as a barrier to PCP. Aspects of the care environment are expected to have the potential to implement [10] and enhance PCP [5]. Therefore, the aspects of the key domain care environment from the PCP framework of McCormack & McCance [9] were chosen to present the results of the current study. The aspects of the key domain care environment are defined in Table 1.

**Table 1:** Aspects of key domain care environment as defined by McCormack (see also Fig. 1) [9]

Power sharing	Power sharing concerns the non-dominant, non-hierarchical relationships that do not exploit individuals, but instead are concerned with achieving the best mutually agreed outcomes through agreed values, goals, wishes and desires
Supportive organisational systems	Supportive organisational systems are systems that promote initiative, creativity, freedom, and safety of persons, underpinned by a governance framework that emphasises culture, relationships, values, communication, professional autonomy, and accountability
Appropriate skill mix	An appropriate skill mix is most often considered from a nursing context and means the ratio of registered nurses (RNs) and non-registered nurses in a ward/unit nursing team. In a multidisciplinary context, it means the range of staff with the requisite knowledge and skills needed to provide a quality service
Potential for innovation and risk-taking	The potential for innovation and risk-taking concerns the exercising of professional accountability in decision-making that reflects a balance between the best available evidence, professional judgement, local information, and patient/family preferences
The physical environment	The physical environment in the healthcare context concerns the balance of aesthetics with function by paying attention to design, dignity, privacy, sanctuary, choice/control, safety, and universal access with the intention of improving patient, family and staff operational performance and outcomes
Effective staff relationships	Effective staff relationships are described as interpersonal connections that are productive in the achievement of holistic person-centred care
Shared decision-making systems^a^	Shared decision-making systems involve the organisational commitment to collaborative, inclusive and participative ways of engaging within and between teams

^a^Sharing decision making on the level of the resident and staff is part of another key domain: person-centred processes

## Methods

### Setting

To examine the policy that board managers of nursing homes developed and implemented to enhance autonomy, two care organisations that aim to enhance autonomy were invited to participate in this study. Both organisations are partner in the Academic Collaborative Centre for Older Adults [11] and were willing to be part in generating knowledge about autonomy. Through studying two organisations the authors aimed to get insight into different policies and thus to collect richer data.

Both organisations provide 24/7 care to older adults. As the current study focuses on the policy to enhance the autonomy of older adults with physical impairments, specific policies for geriatric revalidation units and the psychogeriatric units were not included. One unit from each of the two organisations has previously participated in two earlier studies to gain more knowledge of the perspective of older adults with physical impairments and staff, concerning maintaining and enhancing autonomy [12, 13].

Care organisation A approaches autonomy as follows: ‘autonomy and being active creates happiness.’ This organisation has in total 2700 clients, 2600 employees and 1.150 volunteers. It provides care in 14 locations in a large town in the South of the Netherlands. Organisation B changed the word autonomy into ownership. This was done with the idea that autonomy can be limited or overridden, while persons can and always will be the owner of their life. This organisation has 960 clients, 870 employees and 600 volunteers and provides care in five locations in a small and medium-sized town and surroundings in the same region as Organisation A.

The data about the policies concerning the care environment, aimed at enhancing autonomy, were gathered on the organisational level as well as on the level of the two units that participated in the earlier studies.

### Design

A qualitative descriptive design was chosen to answer the aim of this study using two different methods to collect data: a document study (RQ1-2) and an interview study (RQ 3).

### Document study

To answer RQ 1, which policy is developed by board managers of nursing homes with the aim to enhance autonomy and RQ 2 what is reported in the proceedings and evaluation of this policy, a document study was conducted. In this way it was studied in detail how the policy was planned, discussed, implemented, and evaluated during a period of three years.

#### Inclusion criteria

Documents were selected on two levels: 1) organisational management plans and minutes and other documents regarding the policy towards autonomy enhancement of older adults with physical impairments living in this nursing home and 2) local documents of the two selected units, such as an information booklet about the unit for older adults and leaflets.

#### Data collection

The researcher and first author (JvL), was given access to the active archive by the board secretaries of organisations A and B. Documents were screened for plans to enhance autonomy and the evaluation of the goals mentioned in the plans. For reasons of confidentiality, the researcher was not able to make copies and was not left alone with the documents. However, JvL could make notes and write excerpts. These excerpts were typed out and sent to both corporate secretaries for a member check. They gave written permission to use the summarized content. Some non-confidential documents such as the mission statement about autonomy and detailed plans of specific aspects to enhance autonomy were handed over in print to the researcher by the corporate secretary of both organisations. A contact person from the selected units was asked to provide local documents.

#### Data analysis

Two authors (JvL and IdR) analysed 137 documents (see Table 2) for the policies which were aimed at enhancing autonomy. Both authors had an individual reading of the printed excerpts and the printed documents. They developed and used a data extraction form which consisted of three questions: 1) which policy is described regarding enhancing autonomy 2) is this policy focused on one or more aspect(s) of the care environment (as defined in the PCP framework [9])? and 3) is the information part of a plan to enhance autonomy or is it an evaluation or proceeding of a plan. JvL and IdR wrote down the findings. They separately answered the questions, and subsequently presented and discussed the insights and text fragments to each other in four consensus seeking sessions.

### Semi structured interviews

To answer RQ 3, i.e., what are the perspectives and experiences of stakeholders involved in the development and implementation of the policy in daily practice, semi structured interviews were conducted.

#### Respondents

After receiving permission of the Ethical Review Board of the department of Social and Behavioral Sciences of Tilburg University, no. EC-2017.EX144 and of the Ethical Review Board of organisation A and permission of the board manager of organisation B, stakeholders have been contacted. Purposive sampling was used, by recruiting those respondents who could provide in-depth and detailed information about the development and implementation of the autonomy enhancing policy in the practice of nursing homes [14]. For each organisation, the intention was to recruit ten participants: managers at the strategic, location and the unit level. Furthermore, for each of the seven aspects of the care environment one stakeholder was asked to participate. For example, an educational officer from the HR department concerning if and how employees are trained to support the autonomy of residents (supportive organizational systems) and, in the case of power sharing, representatives of the client council and work council. They were identified by the corporate secretaries. Potential respondents were informed by the interviewers about the aim and design of the study with an information letter which was combined with an informed consent letter. The information letter included a paragraph about asking questions. The name of the contact person and contact information were mentioned. The interview format started with the mandatory topic of asking whether the respondents had any questions and answering them, before signing and handing in the informed consent letter.

#### Data collection

Two nursing students conducted the semi-structured interviews under supervision of the first author. One interviewer conducted all the interviews at organisation A and the second interviewer conducted the interviews in organisation B. They were both not involved in the organisation before and after this study.

To get acquainted with the context of the organisation, the interviewers spent one day on a unit of organization A or B. The first researcher and the interviewers prepared the topic list for the interviews by reading the documents that were collected for RQ 1 and 2. Each topic list was tailored to the interviewed stakeholder based on the aspect(s) of the autonomy enhancing policy the respondent was involved in. For example, the manager was asked how an effective skill mix in the unit was ensured. The member of the client council was asked about the participation in decisions on autonomy enhancing policies in the client council.

Eight respondents from organisation A and nine from organisation B gave written consent for an interview and actually participated. The respondents were interviewed in person in the organisations, one interview took place by telephone because this respondent had no scheduled visits to organisation B on the day of the interview. Each interview was audio recorded and the recordings were transcribed verbatim. The interviews lasted between 7 and 45 min, with a mean of 25 min. The respondent of the 7-min interview was a representative of the residents in the management team, who found it difficult to express reflections on the topics of the interview.

#### Data analysis

Three authors (JvL, BJ and MJ) coded the transcripts [15]. They started with one transcript which they coded independently from each other, followed by a consensus seeking session about the coding of the fragments. They used open coding to code fragments on what they reveal about the policy to enhance autonomy of older adults with physical impairments. After the consensus seeking session, it was decided that two authors coded all transcripts for organisation A as well as for organisation B to follow the process from the development of a policy to how it is implemented and evaluated in each organisation. JvL coded all transcripts of both organisations, MJ coded the transcripts of organisation A and BJ coded the transcripts of organisation B. JvL and MJ had two sessions to discuss the coding of organisation A and JvL and BJ did the same for organisation B. Afterwards BJ, MJ and JvL had a final session to discuss the coding of the fragments [16].

After consensus was reached about the codes, they were processed with ATLAS.ti. After coding was finished, JvL thematised the codes in a deductive way, using ATLAS.ti. JvL established which codes were related to a certain aspect of the care environment of the PCP Framework [9]. MJ checked this step in the process. JvL and MJ discussed codes that could be related to two aspects of the care environment until consensus was reached about which aspect would be the best fit. When in doubt to which aspect of the care environment a code should be attributed, it was discussed until consensus was reached. Codes that referred to other key domains of the PCP Framework, i.e., to person-centred processes and prerequisites of staff, have been assigned to these domains. These codes were not seen as results for the current study and therefore are not discussed in the results below.

## Results

The 137 studied documents, presented in Table 2, consisted of ten non-confidential documents such as multiyear strategy plans and the mission statements on autonomy, and 123 confidential documents such as minutes, i.e., official records of the proceedings of the meetings of the board managers and/or the supervisory board and/or councils. The four local documents concerned for example an introduction of the unit for new residents and newsletters.

**Table 2:** Documents studied for RQ 1 and 2

D = Document code	Regarding	Type of document	Number of documents
**Organisation A**			
DA1	Multiyear strategy plan 2016–2019	Policy document	*N* = 1
DA2	Executive framework 2018 (d.d.12–10-2017)	Policy document	*N* = 1
DA3	Information about living in unit A Version 2017	Information booklet	*N* = 1
DA4	Collected fragments on aspects of the care environment that are related to enhancing autonomy from documents of the executive board (minutes and annexes), of meetings of the executive board with the supervisory board, the work- and client council April 2015- November 2017	Minutes with annexes	*N* = 72
Total A			*N* = 75
**Organisation B**			
DB1	Quick scan and reflection in 2017 The journey to autonomy by client and employee 30–3-2017	Evaluation rapport	*N* = 1
DB2	Minutes of project team 7–4-2016	Record	*N* = 1
DB3	Minutes of the guides 31–5-2016	Record	*N* = 1
DB4	Collected fragments on aspects of the care environment that are related to enhancing autonomy from documents of the executive board (minutes and annexes), of meetings of the executive board with the supervisory board, the work- and client council and the nursing advisory council December 2015- November 2017	Minutes with annexes	*N* = 51
DB5	Plan for a pilot on unit B concerning autonomy	Local plan	*N* = 1
DB6	Newsletter unit B concerning the pilot about enhancing autonomy	Local information	*N* = 1
DB7	Description of a pilot concerning enhancing autonomy on unit B	Local plan	*N* = 1
DB8	Multiyear strategy plan 2015–2018	Policy document	*N* = 1
DB9	Proposal for participation in a national care innovation programme with the autonomy enhancing programme	Organisational plan	*N* = 1
DB10	Factsheet innovation programme concerning autonomy	Public information	*N* = 1
DB11	Progress of the autonomy programme, 2016	Public information	*N* = 1
DB12	Progress of the autonomy programme 2017	Public information	*N* = 1
Total B			*N* = 62
Total			*N* = 137

Table 3 shows the results of the analysis of the above-mentioned documents.

**Table 3:** results of the document analysis

Aspect of PCP	Document type			
	Plan Organisation A to enhance autonomy	Check in minutes, evaluations Local information	Plan Organisation B to enhance autonomy	Check in minutes, evaluations Local information
Power sharing	D^1^A2,DA4: Encourage self-managing teams: employee autonomy and creativity This should lead to resident autonomy	DA4: evaluations of the progress towards self-managing teams, show various interpretations of the concept and goals and a slow progress DA3: living room meetings with residents on the unit	DB4: Encouraging self-managing teams with the expectation that this should lead to desired focus on autonomy of the resident DB4, DB12: Twenty so-called ‘guides’ are installed, and they advise the managers on enhancing autonomy, asked and unasked for DB4, DB9: The work-, resident council and nurses’ advisory council are involved in the plans to enhance autonomy	DB3, DB4: proceedings of the self-managing teams, coaches are appointed for impact self-managing teams DB2,DB3: reports of sessions with guides are seen DB3: ‘guides’ want less to-do lists and more regulation space to enhance autonomy. Such as: to let go of a list on which day a resident can have a shower DB3: behaviour of the ‘guide’ and role of ‘guides’: to rely on, to inspire, stimulate, suggest ideas, challenge, delegate responsibility, disseminate the vision on autonomy DB4: Work council is asked to participate in the new management team DB4: evaluation of actions towards autonomy in the councils
Supportive organisational systems	DA1,DA2: A vision for autonomy has been formulated DA4: Visiting all teams to share the vision on enhancing autonomy	DA3: Vision on autonomy is translated for the unit DA4: the quality indicator is audited: how is the resident maintaining autonomy DA4: Discussion on a culture change for autonomy: how will employees and volunteers do this? Working on autonomy, good communication, not patronising, dignified toilet visits, taking breaks with residents	DB4: Board managers develop a policy directed towards autonomy for the resident DB4: Share the vision on enhancing autonomy via workshops, theatre Theme meetings, workshops, dialogue sessions, autonomy game with the aim that staff is equipped to enhance autonomy Volunteers, therapists, and support services are involved to work together towards autonomy Awareness-raising project to respond to self-managing teams and autonomy DB4: The management team has been expanded with 2 caregivers and 2 care recipients	DB11/DB4: the mangers are ‘drinking coffee’ with the staff of the units in all locations, residents and their families, volunteers, client- work and nursing council and supportive services with the aim to talk about autonomy. The evaluation mentions a movement towards autonomy, although not everywhere in the same pace DB7: the organisation vision is translated to the studied care unit DB9: Coaching and support for the desired dialogue with the resident, training, the resident council is also involved in the training DB5, DB7: dialogues with residents were conducted about their wishes in relation to autonomy DB9, DB10,DB11, DB12: the journey towards autonomy is highlighted with several annual reports on interventions and effects DB3: Training of ‘guides’ is described DB4: the use of an instrument to evaluate the process on the units towards autonomy
Appropriate skill mix			DB4: Sufficiently qualified staff which is related to autonomy Attention to training and retention of staff	DB10: Increased number and training level of staff DB4: concerns about employee mobility and absence from work
Potential for innovation and risk taking	DA2: New dinner concept with more choice in time of the day and menu selection A2, A4: The staff is given more room to experiment with autonomy enhancing activities	DA3: Choice of time and place to eat is mentioned in the local information	DB7: On the selected care unit there is a pilot with an intervention to enhance autonomy DB4, DB8: involving the family in realising autonomy of the resident	DB11: evaluation report by external consultant on the management plans to enhance autonomy. Issues were: - Space versus rules: • What must be done for profession, laws • What is done by organisation B, how does this reflect norms, values and procedures • What can the staff do to translate policy into action - Initially, working with self-managing teams was seen as a mean to enhance autonomy but became a goal in itself - No measurement of progress towards autonomy - Mistakes are repeated - No increase in the satisfaction of residents could be measured yet DB6 Family is expected to contribute to the care of the resident and activities of the resident/unit DB4: new technology is seen as promising for autonomy
The physical environment	DA2, DA4: Plans for new housing concepts: private sanitary facilities, more spacious rooms that provide choice for the older adults with physical impairments that live in a nursing home which should lead to (more) autonomy	DA3: Having an own key to enter the location, unit, and the room	DB4, DB8: Residents can decorate their own apartment. Appropriate living environment to live the life residents want to live	DB11: the evaluation mention that standard offering of curtains and furniture is no longer present
Effective staff relations	DA4: Multidisciplinary collaboration on goals determined by the client		DB8, DB9: Strengthening the collaboration of all staff members with the aim to enhance the autonomy of the resident	DB3, DB4: in the evaluations reciprocity is mentioned as a value in the cooperation DB4: After agreements are made, everyone is responsible to call each other to account. Staff must search for new roles to do so DB9: being inclusive in the actions towards autonomy enhancement: e.g., also giving paramedic and medic professionals a role
Shared decision-making systems				

1 D = document, A/B = organisation A or B, 1–12 = document number

The respondents who participated in the interviews represented departments or councils that were responsible for or involved in one or more aspect(s) of the implementation of the policy to enhance autonomy. Table 4 presents the demographics of the interviewed stakeholders.

**Table 4:** Demographics of the interviewed stakeholders in organisations A and B

	Job title	Years of working in the in current function
A1	Team manager	13
A2	Board manager	6
A3	Human resource management: educational officer	1,5
A4	Member of the work council	^a^
A5	Client advisor	15
A6	Quality and innovation manager	^a^
A7	Senior staff nurse	6
A8	Member of the client Council	1
B1	Occupational therapist	5
B2	Guide	9
B3	Human resource management: educational officer	0,5
B4	Location manager	2,5
B5	Team coach concerning autonomy enhancing	1
B6	Board manager	8
B7	Facility manager	^a^
B8	Paramedic professional	1,5
B9	Representative of the residents in the management team	2

^a^Missing values

In Table 5, the codes, and their allocation to the aspects of the care environment are shown.

**Table 5:** Codes from interviews A and B

Aspects derived from PCC	Codes of organisation A	Respondents *N* = 8 Followed by fragments	Codes of organisation B	Respondents *N* = 9 Followed by fragments
Power sharing	Organisation of participation: client council (6 fragments)	A8:6	Organisation of participation: client council, management team extended with clients and advised by guides, bottom-up signals (13 fragments)	B2:1, B4:5, B6:3, B9:4
	Participation in decisions of daily life/ living together: living room meetings in the unit (1 fragment)	A1:1	Participation in decisions of daily life/ living together: living room meetings in the unit (3 fragments)	B2:2, B9:1
	Self-managing teams (3 fragments)	A1:1, A2:1, A3:1	Self-managing teams (1 fragment)	B6:1
Supportive organisational systems	Offering training, tools, coaching (9 fragments)	A1: 2, A2:2, A3:1, A4:1, A6:1, A7:2	Offering training, tools, coaching (16 fragments)	B1:2, B3:1, B4:1, B5:7, B6:5
	Establishing structures for: regulatory requirements, quality measurements, coaches, work schedules (5 fragments)	A1:1, A6:3, A7:1	Establishing structures for: regulatory requirements, quality measurements, coaches, guides, role models, organisational consultation structure (27 fragments)	B1:4, B2:6, B4:2, B5:6; B6:5, B8:3, B9:1
	Role managers: coach, policy making, intervene in case of discrepancies (7 fragments)	A1:1, A2:2, A3:1, A6:1, A7:1, A8:1	Role managers: support, coach, basis in order, intervene in case of discrepancies (21 fragments)	B2:2, B4:4, B5:8, B6:7
	Define vision and core values, communicate these to staff and clients, evaluate these and live by these (19 fragments)	A1:2, A2:5, A3:4, A4:1, A5:3, A6:3, A8:1	Define vision and core values, communicate these to staff and clients, evaluate these and live by these (31 fragments)	B1:2, B2:4, B3:1, B4:9, B5:5, B6:8, B7:1, B8:1
Appropriate skill mix	Team composition: Mix of expertise/ well-trained (4 fragments)	A1:2, A2:1, A3:1	Team composition: Mix of expertise/ well-trained (11 fragments)	B4:2, B5:2, B6:2, B7:1, B8:4
	Sufficient permanent staff: not always possible, turnovers, effort to become a team again and again (12 fragments)	A1:2, A2:1, A4: 1, A7:7	Sufficient permanent staff: not always possible, turnovers, effort to become a team again and again (7 fragments)	B1:1, B2:1, B4:3, B5:2
Potential for innovation and risk taking	Organisational culture: the courage to develop a policy to enhance autonomy in the face of cut government budget (1 fragment)	A2:1	Organisational culture: the courage to develop a policy to enhance autonomy in the face of cut government budget (2 fragments)	B6:2
	Space to regulate: being allowed to make mistakes, to develop, to take initiatives, to plan duty rosters themselves, to budget. (16 fragments)	A1:1, A2:6, A3:1, A6:2, A7:7	Space to regulate: being allowed to make mistakes, to develop, to take initiatives, to plan duty rosters themselves, to budget. (21 fragments)	B1:3, B2:5, B3:2,B4:2, B5:2, B6:5, B8:2
	Expectations of care technology: maintaining autonomy (1 fragment)	A6:1	Expectations of care technology: ADL, electronic care record, medication administration, mobility (5 fragments)	B4: 4, B6:1
	Increasing choice: when to get up, when to eat, when to drink, choice for activities (12 fragments)	A1:4, A2:1, A5:2, A6:2, A7:1, A8: 2	Increasing choice: when to get up, when to eat, when to drink, choice for activities (15 fragments)	B2:6, B3:1, B6:2, B7:5, B9:1
The physical environment	Adjusting to needs (6 fragments)	A1:2, A2:2, A5:1, A7:1	Adjusting to needs (5 fragments)	B1:3, B3:1, B4: 1
	Increase freedom of choice: decorate own room (2 fragments)	A5:1, A7:1	Increase freedom to go outside: do residents have an own or lend key to enter the building (1 fragment)	B2:1
	Limited choice to move to a preferred nursing home due to waiting lists (1 fragment)	A5:1		
Effective staff relationships			Dilemma as a staff member: working together as a team, working with a manager who does not want to go along with the policy? (3 fragments)	B1:1 B2:2
			Role of managers: do they actually want to share power? (2 fragments)	B2:2
Shared decision- making systems	Setting boundaries: professional code (1 fragment)	A5:1	Setting boundaries: professional code (2 fragments)	B6:1, B8:1

The overarching research question was which policy, aimed to enhance the autonomy of older adults with physical impairments in nursing homes, is developed and implemented. The results will be presented following the aspects of the key domain care environment of the PCP framework (see Table 1) [9]. Per aspect, the results are structured as follows: the intended policy as described in the documents, proceedings and evaluation as described in the documents and the perspectives and experiences as shared by the interviewed respondents involved in the implementation of the policy.

### Aspect 1 power sharing

Four policies were found in this aspect: i.e., the development towards self-managing teams, installing role models, participation from the councils and living room meetings and access to, and involvement in, the care plan.

#### The development towards self-managing teams

It was read in the documents, that the board managers of both organisations planned to approach the autonomy of residents indirectly with a policy to implement self-managing teams. These teams should provide care on a unit in a more autonomous way. In the plans of both organisations, it was claimed that self-managing teams would lead to more focus on autonomy of older adults living in the nursing home. In the minutes, a development of the teams in both organisations towards self-managing with a manager as coach, was found. The progress of the policy was regularly discussed by the board managers of both organisations with the supervisory boards and the councils. However, it should be noted that the discussion was merely limited to team development, and it was not related to enhancing autonomy of older adults. In the interview, respondent A2 put autonomy at the heart of the development of self-managing teams.

Respondent A2 said: ‘I think that if you want to give autonomy a place, value it. You will have to create a context for it in the staff on the units. That is where the focus is now. We work with self-managing teams and independent thinking professionals who are attuned to the client.’

#### Installing role models

In the plans and proceedings of organisation B, it was found that the management team was supported by 20 so-called ‘guides’ working in the teams. Guides were meant to have the responsibility to pioneer in activities towards enhancing autonomy of residents (role models). In the documents of organisation B, it could be read that the guides were in position. However, in the proceedings of the meetings of the guides, issues concerning responsibilities of the guides were found. It was read that they asked themselves ‘how far can we go when acting outside the box’? One interview was with respondent B2 who was one of these guides. The guide mentioned that ‘thinking out of the box’ and challenging the team was not appreciated by the team manager. On the contrary, respondent B6 mentioned that the board manager wanted to welcome bottom-up signals to the top and wanted to have direct feedback on plans from residents and staff in the management team.

#### Participation of work and client councils and living room meetings

In the minutes of both organisations, it was found that they had the legally required participation bodies such as a client council and a work council. Moreover, organisation B also had the recommended nursing advisory council. In the minutes of the board managers, it was read that the councils in their regular meetings with the board were consulted and asked for consent on the topic of enhancing autonomy of residents. Furthermore, it was read in the documents of organisation B, that members of the client council and work council participated in a training to enhance autonomy. This was a dialogue training to start the conversation with the client about autonomy, managers were trained to place the client at the centre.

In the local document of organisation A, it was found that power sharing on the unit level was implemented by living room meetings between residents and staff on the unit. In the interviews, the living room meetings were mentioned several times as a way to participate in decision making about daily life on the unit in both organisations.

Respondent B9 said: ‘once in a while, we have a meeting with everyone in the unit. For example, we talk about mealtimes, whether everyone still agrees with the times of the meals or whether the time should be changed. [Also, about] the location of the meals.’

#### Access to and involvement in the care plan

In the documents of both organisations, plans and proceedings were found about the older adult’s access to their electronic care plan. Furthermore, references were found to protocols to ensure residents could be present in scheduled meetings to evaluate their care plans. In the minutes of both organisations, a follow-up of the proceedings of the access to the electronic care plan and the implementation was found. In the interview respondent B4 expressed that a further expansion of the access to the care plan towards a resident’s full ownership could enhance autonomy in the future.

Respondent B4 said: ‘my ultimate goal is that every resident has his own tablet. And that he is the owner of his own device and of his information and that we log in to his device. And not as it is now that he logs in with us but that it really is his [care plan].’

### Aspect 2 supportive organisational systems

It was found in the documents that a corporate vision on autonomy was formulated and communicated on the website and other public media by both organisations. The board managers of organisation A visited all locations and shared their vision with the staff on autonomy enhancement for the residents. The board manager of organisation B shared the vision with the staff via workshops and theatre and visited locations as a follow-up. Furthermore, organisation B offered coaching, an autonomy game, and annual updates. Moreover, the management team of organisation B was expanded with two representatives of the residents and two of the caregivers. In the proceedings, it was found that the quality department of organisation A did an internal audit on autonomy enhancement and organisation B measured and evaluated the planned policy itself. However, there was no evaluation found in the minutes whether the autonomy of residents was enhanced. The interviewed respondents recognised the activities the organisation used to enhance autonomy in daily practice. They mentioned that one could learn and share experiences about enhancing autonomy inside and outside the organisation. Role models were appointed to enhance autonomy. Respondents stated that organisation A offered no special training; the vision on autonomy was merely communicated by the organisation and new employees were informed. The respondents in organisation B mentioned that training, tools, and coaches were available for staff to enhance autonomy of older adults. Furthermore, respondents mentioned that residents and nurses were included in the management team with the aim to strengthen the policy towards autonomy. The vision on autonomy was known by the respondents of both organisations and they tried to comply to the vision.

Respondent B6 said: ‘we have also set up a whole training programme. We have a number of workshops about autonomy, how to have a dialogue [with residents], what are the key moments in care, and when I say care, I mean (.) in the contact with a resident. That is constantly repeating, repeating, repeating, repeating. The good examples and also the things that aren't going well, with the purpose to learn from each other’.

### Aspect 3 appropriate skill mix

No specific documentation regarding policies concerning skill mix to enhance autonomy were found in Organisation A. The policy of organisation B focussed on recruiting more staff and BN’s. This was expected to enhance autonomy. In the minutes of organisation B concern was read about the discontinuity of care because of interim staff. In a factsheet of organisation B concerning the progress of the policy towards autonomy enhancement, an increase in the number of staff members in the nursing home and their educational level was described. In the interviews, respondents mentioned planning problems, because there was not sufficient and permanent staff. In terms of staff composition, the team needs to be competent in enhancing autonomy. New employees should be educated and able to fit in. But this appeared not to be the case. Respondents expressed they had ‘to start all over again’ to talk about the vision on autonomy when new staff was recruited. According to the respondents, nursing schools should change the curriculum regarding enhancing autonomy. The organisational aim to have a balanced team composition with a mix of expertise was known by the respondents of organisation B. However, the objective of the policy to have more BN’s was not clear for the respondents. Respondent B5 expressed concerns that the team was more involved with the new roles of the team members after BN’s were recruited, than with the autonomy of older adults.

Respondent B5 said: ‘We used to have the auxiliary nurses as care coordinators. Then later on we got nurses with a bachelor’s degree, and they became the care coordinators for residents, so that was already a bit awkward, but you could still explain that residents (…) needed more serious care (…). The BN sat almost on the chair of the team manager. And then you have two captains on one ship. And that in a team that has to enhance autonomy’.

### Aspect 4 potential for innovation and risk taking

In the aspect potential for innovation and risk taking three policies were found: innovations towards autonomy enhancement in a financial difficult time, choice enhancing policies and expectations from autonomy increasing technology.

#### Innovations towards autonomy enhancement in a financial difficult time

The board manager of organisation B wrote explicitly in the plans that it is understandable that in such a learning process towards autonomy, mistakes can be made and should be allowed. Some respondents said that, given the conditions of a cut back of budgets on nursing homes by the government, it took courage and motivation of the management to start a programme to enhance autonomy. Prerequisites, such as time and space, to develop competences to enhance autonomy were arranged. Staff could take initiatives such as letting go of fixed times of care moments. However, the respondents expressed their concerns about the consequences of this freedom on the level of the units: financial problems, problems with scheduling, cooperation and employees who create their own work activities.

#### Choice enhancing policies

It was read in the documents that both organisations created opportunities for choice and preferences of the resident e.g., they both aimed to enhance choice through a new meal system. Choice was supposed to be an act of autonomy. The respondents of organisation A mentioned an increasing freedom of choice for the residents in the daily schedule, choice regarding eating and drinking, getting up at a preferred time and choosing activities.

Respondent A6 said: ‘autonomy can express itself in daily activities such as washing, dressing, and eating. Let's talk about food. If someone wants vegetarian food, I think we should think about how to organise that for that person. That is important to him for now.’

#### Expectations from autonomy increasing technology

In the minutes of organisation B new technologies within the nursing home, were considered as promising for autonomy, such as technology that supports activities of daily living (ADL) and mobility. One respondent of organisation A said that technology could be valuable for older adults to enhance autonomy. Respondents of organisation B also expected much from technology to enhance autonomy for the assistance in ADL, mobility, the day structure and independently taking medication with a medicine dispenser. Although a lot was expected, no information about the implementation was found.

### Aspect 5 the physical environment

In the documents, the physical environment as a means to enhance autonomy was reflected in the planned policy towards the interior, furnishing of the rooms and accessibility of the building. The board managers of organisation A had plans for a more suitable living environment for residents in the future: the current building still had shared bathrooms and the rooms were small, which was not considered as an autonomy enhancing environment. However, an actualisation of these plans was not found in the documents. Organisation B planned to vacate the rooms empty. New residents could furnish it themselves, which was seen as autonomy enhancing because they could, for example, choose which furniture was taken from home to decorate the room. In the minutes, it was found that this policy was realised. In the local document of organisation A, it was read that the older adults possessed a key to independently enter the location, the unit, and their private room. The physical environment was mentioned in the interviews in relation to increasing the freedom of choice. The respondents of the interviews confirmed the policy about furnishing the room (organisation B), the advantages of owning a key (organisation A and B) and a better adaptation of the rooms to the needs of older adults (organisation A). A respondent of organisation A mentioned that the existing building had a negative impact on achieving autonomy.

Respondent A7 said: ‘the rooms are very small as you can see, there is no possibility to make coffee or tea. They always depend on when we serve in the living room.’

### Aspect 6 effective staff relations

In the plans of organisation A, it was described that a better collaboration within multidisciplinary teams towards the goals, set by the residents, was needed. The policy of organisation B was aimed at all the professionals working in the nursing home. The monitoring of the commitments, made in the process towards strengthening autonomy, should enhance relations between staff. They should work based on equality, towards autonomy of the residents. In the documents it was found that autonomy enhancement should not only be the responsibility of the staff on the unit but also of the other professionals, such as the facility department. In the interviews effective staff relations were hardly mentioned. One respondent mentioned the difficult collaboration with the manager when trying to be a role model for enhancing autonomy. Respondent B1 mentioned the slow development in the collaboration within the multidisciplinary team towards the autonomy enhancement of the residents.

Respondent B1 said: ‘we try to keep building as a [multidisciplinary] team so that in the end it all benefits the resident. But I think that if you are a team and you are there for each other, you can also be there for the resident. But we are not that far yet.’

### Aspect 7 shared decision-making systems

Shared decision making systems as ‘ways of engaging within and between teams’ [9] are only mentioned in a few interviews, no specific policy was found in the document research.

Shared decision making was brought up in the context of possible conflicting views of professional staff and managers about the residents’ autonomy. One respondent stated that the ‘professional code’ of the health care professionals could easily take precedence over the autonomy of the older adults. B6 declared to choose for the residents in this circumstance.

Respondent B6 said: ‘we collaborate with professionals here: assistants, carers, nurses, therapists, doctors. They all have professional ethics. Yes, and we do say that, if a resident says I do not want medication, I do not want treatment or I do not want that, they can have an opinion from the perspective of their professional ethics. But ultimately, we choose for that resident’.

## Discussion

This study aimed to answer the overarching question: which policy, aimed to enhance the autonomy of older adults with physical impairments in nursing homes, is developed and implemented. The results were organised in the PCP framework. The care environment is one of the key domains of this framework and consists of seven aspects [9]. The results showed that all seven aspects were, to a greater or lesser extent, found in the documents and/or interviews with the respondents. There seems to be a gap between the policies towards enhancing autonomy and the day-to-day practice in the organisations. In general, it can be argued that the intentions and policies at the top of the organisation are ambitious, but the policies are not holistic, often not supported by knowledge and often indirect. Furthermore, the policies don’t seem to be implemented or fully known in the basis of the organisation.

Aspects of the care environment that seem easy to adjust with policies are dominantly addressed by organisations. More permanent aspects, such as the physical environment, receive less consideration. Most policies were directed at the aspects power sharing and supportive organisational systems.

Regarding the aspect of power sharing, there are two notable insights. First, in both organisations it was assumed that an intervention that is indirectly aiming to enhance autonomy, such as self-managing teams would lead to more autonomy of residents. Although it is known that teams with little freedom to regulate opt for rules and safety rather than preferences of older adults [17], there is no evidence for the opposite, i.e. whether self-managing teams will lead to enhanced autonomy of residents. The development of self-managing teams often originates from the ambition to create more organisational flexibility through increasing employees' responsibility and autonomy [18]. Autonomy for staff, however, is not only associated with the practice in the care unit, but also with decision making in the organisations and the way work is organized itself [19]. The last two aspects i.e., decision making in the organisation and organising work itself, seem to be more prevalent in the organisations, where staff was more concerned with coordinating tasks and work, rather than with enhancing residents' autonomy.

Second, considering power sharing, the access to the electronic care plan by older adults is used as an autonomy enhancing policy in both organisations. In practice, few older adults in nursing homes have their own devices and access is often delegated to family members [20, 21]. Equal access to information is important to enhance autonomy, but the policy was a means to itself of which it was not clear whether it contributed to the goal of power sharing.

Concerning the aspect innovation and risk taking it was seen that organisations made finances and time available for innovations to enhance autonomy and thus took financial risks to address the subject of autonomy. This showed a strong commitment that the board managers were willing to make a real change in the organisation. One of the innovations was that staff could let go fixed times for care and stop completing checklists. This led to tensions between staff members on the units and uncertainties in the teams about the finances, responsibilities, and scheduling in the unit. The structures within the care environment are often criticized as influencing autonomy in a negative way [4]. However, when staff is given space to flexibly deal with changing schedules and using checklists, it also requires a certain determination of them to use this freedom [22].

Earlier research already showed that organisational policies mostly concerned choice enhancing or control enhancing policies [7, 8]. This narrow interpretation of autonomy is also seen in the documents and interviews of the current study. Choice enhancing mechanisms in the policies directed at innovation and risk taking and the physical environment, such as innovations in the meal system and the furnishing of the rooms by residents, were found. These identified choice enhancing policies were consistent with the policies found in the study of Sikorska-Simmons [7].

Control enhancing policies were found to be directed at the physical environment, such as having an own key of the building and the residents’ apartment. Control enhancing was found as well in power sharing, i.e., the participation of residents in client councils, living room meetings and the management team (MT) was realised. This participation of several older adults in formal decision-making went beyond mandatory representative bodies such as client councils. However, whether participation of residents in the MT is a suitable policy is discussed by Abma and Baur [8], who identify the risk for tokenism and frictions between the lifeworld of the older adult and the system-world of the MT/organisation. Residents’ participation in collaborative actions in the nursing home is seen as a more effective way to realise power sharing by these authors [8].

The PCP framework indicates that all aspects of the care environment are important to develop a person-centred practice. In this study, the authors found that that there was an overrepresentation of two aspects i.e., power sharing and supportive organisational systems. The authors recommend a more balanced use of all aspects in the care environment in order to create a more autonomy enhancing care environment for older adults in nursing homes [10]. In the PCP framework, the care environment is situated as a key domain between two other key domains i.e., prerequisites of staff and person-centred processes. In the interviews, the respondents referred to these domains by spontaneously sharing some experiences how, in caring for the older adults -the person-centred processes- they explored a way to put autonomy into practice. These expressions also gave an insight into the involvement -prerequisites of staff- of the respondents in autonomy enhancement and the importance of the other key domains as well in enhancing autonomy.

Although the PCP framework poses that all aspects of the key element care environment are important to develop a person-centred practice [9], this is not yet the case in practice. The authors therefore recommend using all seven aspects of the care environment in a balanced combination with the other key domains of the PCP framework to achieve person-centred practice and as a result an enhancement of residents’ autonomy.

### Strengths and limitations

Lincoln and Guba, as cited in Korstjens and Moser, suggest credibility as a quality criterium for qualitative research [23].The following aspects of credibility were taken into account to heighten the trustworthiness of the study.

A strength of the study is that data triangulation, the use of multiple data sources, has been applied [23]. An extensive document study was conducted and besides an analysis on the plans to enhance autonomy, evaluation reports, quarterly reports and annual reports were additionally studied. Also, an analysis was done on local documents in two nursing units. To get more insight into how the policy is known and implemented in the organisation, interviews were conducted in addition to the document analysis. With this triangulation the trustworthiness of the study was strengthened [23]. 

Another strength is that the respondents for the interviews were purposively selected. One or two stakeholders were interviewed about one or more aspects of the care environment they were involved in [14]. However, this could have resulted in a response bias. In some cases, the respondents started guessing, improvising or expressing resentments because they did not know an answer to the question [24]. These fragments in the transcripts were not used. The authors also aimed to include the voice of a representative of the residents in the management team about the experienced power sharing in this study. However, the authors realise that a semi-structured interview was not the best method to include resident's voices. Nevertheless, the resident did give an insight into the implementation of the power sharing policy that focused on participation of representatives among the residents. Furthermore, the resident considered the contribution to the management team as valuable, even if it was difficult to articulate what was important in that regard.

Another strength is that the board managers of both organisations allowed the authors to use confidential sources to increase the insights about how both organisations aim to enhance the autonomy. A limitation can be that these confidential documents were studied by one researcher, who could only take notes. This could have led to bias. To prevent this, the notes were typed out and presented to the corporate secretary of each organisation. Through this member check, permission was asked and given to use the checked confidential information in the study. Moreover, non-confidential documents were available and could be copied and entirely analysed by two authors independently from each other.

A limitation can be that the interviews were conducted by fourth year BN students who were inexperienced in interviewing. However, the first researcher who has experience in interviewing and qualitative research methods, guided the interviewers during the data collection. Moreover, an expert in the field of the PCP framework and autonomy enhancement of the university of applied science, supervised these students.

A last strength is the use of investigator triangulation. The data extraction of the documents and interviews was done in pairs. After individual coding, a discussion in pairs followed, whereafter consensus meetings were held [23]. The authors also found consensus on the allocation of the codes and fragments to in the different aspects of the key domain care environment or to other key domains of the PCP framework.

### Recommendation for further research

As the current study was directed at the organisational perspective, the researchers did not ask older adults what changes in the care environment they would propose to exert more autonomy, nor was the impact of the policy on the autonomy of the older adults themselves studied. It is recommended to study aspects in the care environment that are considered as urgent or important, by older adults living in nursing homes. This can be done with a participative action research design: actions toward the enhancement of autonomy chosen by the older adults can be explored and followed by reflection, to bring about a change in the care environment [25]. Furthermore, if researchers want to include the voice of older adults into research on autonomy enhancement, research methods tailored to the condition of older adults, will be needed. Such as creative materials that help articulate the residents voice better [26].

### Implications for practice

The insights about policies to enhance the autonomy of older adults with physical impairments as found in the current study can provide guidance for the planning of new or current policies. The actual policies that are being implemented in organisations can be compared with the policies as described in this study.

Several lessons were learned in this study. First, it is advised to develop a holistic policy, that in a balanced way is related to all the aspects of the care environment. Second, it is of utmost value to consider both the perspectives of older adults and staff. Third, attention should be paid to supporting and training staff in implementing the policy. Staff certainly needs new skills e.g., how to navigate between rules, routines, procedures, and the life world of residents. An example is thinking about how coffee and tea facilities could be made available to residents. In this way, residents are able to drink coffee or tea whenever they like (they have a choice) and also, they can offer their visitors something to drink. Fourth and last, it is important to be clear what the expectations are, about enhancing autonomy to those involved. For example, when organisations opt for implicit or indirect improvement of autonomy through a team intervention such as self-managing teams, it is advised to set goals, use interventions such as coaching for the older adults as well and evaluate the impact on autonomy enhancement of older adults. Another example is a policy aimed at recruiting specific staff, such as bachelor nurses. It should be clear to them, residents and other staff members what is expected of this specific role, responsibilities, and expectations with regard to autonomy enhancement.

## Data Availability

The datasets generated and/or analysed during the current study are not publicly available due to all respondents were informed that the data gathered would only be used and read by the researchers and will not be given to third parties. Moreover, the dataset consists of qualitative Dutch data, but are available from the corresponding author on reasonable request.

## Abbreviations

PCP: Person-centred practice
ADL: Activities of daily living
BN: Bachelor Nurse
